# Catalogue of the Medical Library of Pennsylvania Hospital

**Published:** 1857-11

**Authors:** 


ART. IX.—*Catalogue Raisonné of the Medical Library of the Pennsylvania Hospital.* By EMIL FISCHER, M.D. Printed by Order of the Board of Managers. T. K. & P. G. Collins, Printers. Philadelphia. 1857. Octavo, pp. 750, including Index of Authors' Names.

IF a good library be a great boon to the student, whether pupil, expert or preceptor, the next best thing for all concerned is, certainly, a well-furnished and well-digested catalogue. The medical students of Philadelphia have long enjoyed the former in the well-known collection of the Pennsylvania Hospital; and they may now revel in the latter, since they have, at last, within their reach, a catalogue, which, in every use and characteristic of such a guide, is far in advance even of the large and excellent library, which it so admirably represents.

This library is said, we believe justly, to be the best of its kind, as it is the first of its date, in the country; but whether the pre-eminence thus claimed be admitted or not, there can be no question whatever as to the superiority of the catalogue just issued by the Hospital managers. It is not excelled, if equalled, by any yet published in the English language; and will safely bear comparison with similar productions in other European tongues.

Although preceded, at various periods, since the foundation in 1763, by three different compilations, each of which was subsequently augmented with a supplement, it has been prepared independently of these, and is, therefore, an entirely new work.

"For the old plan," says Dr. Fischer, "of disposing the works in the alphabetical order of the names of their authors, a classified arrangement, according to subjects, has been substituted. The advantages of a catalogue raisonné are so evident that it is hardly necessary to particularize them. Not only the student who is anxious to familiarize himself with the depth and breadth of medical literature, but also the medical writer in his laborious research for authorities, which he might consult on the subject of his investigation, will derive material aid from a work of this kind.

"For the classification used in the present catalogue, the compiler is essentially indebted to the catalogue raisonné of the library of the Medical Society of Edinburgh, the arrangement of which has been followed out as far as some difference in the compass and character of the two libraries would permit.

"The whole work has been divided into four parts, viz., Medicine, Science, Literature, and Miscellanies. Each part has been subdivided into chapters, the first and most important part containing eight of them, viz.: I. Anatomy, including Human and Comparative Anatomy; II. Physiology; III. Materia Medica and Pharmacy, including Hygiene and Therapeutics; IV. General Pathology, and Practice of Medicine; V. Surgery; VI. Midwifery, and Diseases of Women and Children; VII. Medical Jurisprudence and Medical Police; and, VIII. Medical Literature.

"It will be found that the classification and subclassification of the different chapters coincide, as far as can be in a work of this kind, with those adopted in most systematic works on the different branches of medical and general science. Only in those instances where no scientific ground for classification could be found the alphabetical arrangement has been had recourse to.

"For the disposition of a book, not only its title, but also its contents had to be taken into consideration. In order not to separate a work from others contained under a certain subhead, and treating on the same subject, but from different points of view, its relation to the general head had to be sometimes disregarded.

"Titles which had to be repeated frequently, were abbreviated, in accordance to the head under which they were placed, and by this measure unnecessary incumbrance of the catalogue was avoided. The space thus gained has been taken advantage of for the quotation of monographs, essays, &c., contained in collective works, or in appendices to larger treatises. These quotations have been necessarily confined to such essays, which either were known to have been published in a separate form, or which, from the nature of their contents or extent, seemed to be of some importance. Some of these points had to be ascertained by the aid of bibliographical works."

The foregoing extract, from his introduction, will afford some idea of the great value of Dr. Fischer's compilation, not merely to the frequenters of the library, but as a means of reference even to those who have no direct access to the volumes which it indicates. From it also, those, at least, who have some knowledge of such undertakings, may form a faint conception of the obligation we are all under to its untiring and able author, for the vast amount of manual and intellectual labor which he has so successfully expended in the preparation of its pages, and in their supervision through the press.

The thanks of the whole profession are due also to the Managers of the Pennsylvania Hospital, for the wise liberality displayed in the publication of so honorable and instructive an exponent of their noble library; and we cordially congratulate them, not only on their fortunate selection of Dr. Fischer for the work, but on the very handsome manner in which they have allowed the printers to present it to the public.

We sincerely hope, for the sake of all who resort to Philadelphia, or reside here, in pursuit of medical knowledge, that Dr. Fischer may early and abundantly enjoy the reward, which alone he asks for, in the increasing usefulness of the library, and the augmenting interest manifested in its treasures by the medical fraternity, for whose advantage it was originally founded, and has been so long and so efficiently fostered.

Copies may be obtained from the librarian at the Hospital.

